# Validity and reliability of a pilot scale for assessment of multiple system atrophy symptoms

**DOI:** 10.1186/s40673-017-0067-5

**Published:** 2017-07-03

**Authors:** Masaaki Matsushima, Ichiro Yabe, Ikuko Takahashi, Makoto Hirotani, Takahiro Kano, Kazuhiro Horiuchi, Hideki Houzen, Hidenao Sasaki

**Affiliations:** 1 Department of Neurology, Faculty of Medicine and Graduate School of Medicine, Hokkaido University, Kita-15, Nishi-7, Kita-ku, Sapporo, 060-8638 Japan; 2 Department of Neurology, Obihiro Kosei Hospital, Nishi-6, Minami-8, Obihiro, 080-0016 Japan

**Keywords:** Multiple system atrophy, Symptom assessment scale, Intraclass correlation coefficient, Cronbach’s α coefficient

## Abstract

**Background:**

Multiple system atrophy (MSA) is a rare progressive neurodegenerative disorder for which brief yet sensitive scale is required in order for use in clinical trials and general screening. We previously compared several scales for the assessment of MSA symptoms and devised an eight-item pilot scale with large standardized response mean [handwriting, finger taps, transfers, standing with feet together, turning trunk, turning 360°, gait, body sway]. The aim of the present study is to investigate the validity and reliability of a simple pilot scale for assessment of multiple system atrophy symptoms.

**Methods:**

Thirty-two patients with MSA (15 male/17 female; 20 cerebellar subtype [MSA-C]/12 parkinsonian subtype [MSA-P]) were prospectively registered between January 1, 2014 and February 28, 2015. Patients were evaluated by two independent raters using the Unified MSA Rating Scale (UMSARS), Scale for Assessment and Rating of Ataxia (SARA), and the pilot scale. Correlations between UMSARS, SARA, pilot scale scores, intraclass correlation coefficients (ICCs), and Cronbach’s alpha coefficients were calculated.

**Results:**

Pilot scale scores significantly correlated with scores for UMSARS Parts I, II, and IV as well as with SARA scores. Intra-rater and inter-rater ICCs and Cronbach’s alpha coefficients remained high (> 0.94) for all measures.

**Conclusion:**

The results of the present study indicate the validity and reliability of the eight-item pilot scale, particularly for the assessment of symptoms in patients with early state multiple system atrophy.

## Background

Multiple system atrophy (MSA) is a rare progressive neurodegenerative disease characterized by autonomic dysfunction, Parkinsonism, and ataxia [1, 2]. MSA patients generally need wheelchairs in five years and die in ten years from disease onset. Though some underlying mechanisms of MSA have been revealed, such as the aggregation of α-synuclein to oligodendroglia, the complete pathogenesis of the disease remains to be elucidated [3]. As quantitative biomarkers for MSA have not yet been developed for use in clinical trials, clinicians must rely on evaluations of changes in symptoms. However, the usefulness of such evaluation varies according to the scale used, and the large numbers of patients required for MSA trials render redundant and unresponsive scales impractical. Therefore, a brief yet sensitive scale is desirable for clinical trials involving patients with MSA.

In a previous study, we compared the following five scales in their ability to assess symptoms of MSA [4]: Unified MSA Rating Scale (UMSARS) [5], Scale for the Assessment and Rating of Ataxia (SARA) [6], Berg Balance Scale (BBS) [7], MSA Health-Related Quality of Life scale (MSA-QoL) [8], and Scales for Outcomes in Parkinson’s Disease–Autonomic Questionnaire (SCOPA-AUT) [9]. We subsequently devised a simple pilot scale comprised of eight items representative of those exhibiting the largest standardized response means (handwriting, finger taps, transfers, standing with feet together, turning trunk, turning 360°, gait, and body sway) [4].

Our prior study revealed that the UMSARS Part II (motor examination), Part IV (global disability scale, SARA, and BBS are effective in evaluating MSA progression over 12 months, indicating their potential to assess rapid changes in MSA symptoms. Detailed item-by-item analyses suggested that the largest SRMs were obtained for the following items: handwriting, finger taps, transfers, standing with feet together, turning trunk, turning 360 degrees, gait, and body sway. Further analyses revealed that our eight-item semi-quantitative (total score = 36 points) pilot scale (Table 1) exhibited an SRM larger than those observed for the UMSARS Part II/Part IV, SARA, and BBS [4], suggesting that the pilot scale was most effective in detecting rapid changes in symptoms of MSA. In the present study, we aimed to investigate the validity and reliability of the pilot scale for the assessment of symptoms in patients with both cerebellar and parkinsonian subtypes of MSA.

**Table 1:** The items of the pilot scale

1. Gait (from SARA 1)
Patient is asked (1) to walk at a safe distance parallel to a wall including a half-turn (turn around to face the opposite direction of gait) and (2) to walk in tandem (heel-to-toe) without support.
0. Normal, no difficulties in walking, turning, or walking in tandem (up to one misstep allowed)
1. Slight difficulties, only visible when walking 10 consecutive steps in tandem
2. Clearly abnormal, tandem walking >10 steps not possible
3. Considerable staggering, difficulties in half-turn, but without support
4. Marked staggering, intermittent support of the wall required
5. Severe staggering, permanent support of one stick or light support by one arm required
6. Walking >10 m only with strong support (two special sticks or stroller or accompanying person)
7. Walking <10 m only with strong support (two special sticks or stroller or accompanying person)
8. Unable to walk, even if supported
2. Transfers (from BBS 5)
Arrange chairs(s) for a pivot transfer. Ask patient to transfer one way toward a seat with armrests and one way toward a seat without armrests. Two chairs (one with and one without armrests) or a bed and a chair may be used.
0. Able to transfer safely with minor use of hands
1. Able to transfer safely definite need of hands
2. Able to transfer with verbal cueing and/or supervision
3. Needs one person to assist
4. Needs two people to assist or supervise to be safe
3. Finger tapping (from UMSARS Part II–8)
Patient taps thumb with index finger in rapid succession with widest amplitude possible, with each hand for at least 15 to 20 s. Rate the worst affected limb. Note that impaired performance on this task can be caused by bradykinesia and/or cerebellar incoordination. Rate functional performance regardless of underlying motor disorder.
0. Normal.
1. Mildly impaired.
2. Moderately impaired.
3. Severely impaired.
4. Can barely perform the task.
4. Handwriting (from UMSARS Part I–3)
0. Normal
1. Mildly impaired (all words are legible).
2. Moderately impaired (up to half of the words are illegible).
3. Markedly impaired (the majority of words are illegible).
4. Unable to write
5. Standing unsupported with feet together (from BBS 7)
Place your feet together and stand without holding
0. Able to place feet together independently and stand 1 min safely
1. Able to place feet together independently and stand for 1 min with supervision
2. Able to place feet together independently but unable to hold for 30 s
3. Needs help to attain position but able to stand 15 s feet together
4. Needs help to attain position and unable to hold for 15 s
6. Turning to look behind over left and right shoulders while standing (from BBS 10)
Turn to look directly behind you over toward left shoulder. Repeat to the right. Examiner may pick an object to look at directly behind the subject to encourage a better twist turn.
0. Looks behind from both sides and weight shifts well
1. Looks behind one side only other side shows less weight shift
2. Turns sideways only but maintains balance
3. Needs supervision when turning
4. Needs assist to keep from losing balance or falling
7. Turning 360° (from BBS 11)
Turn completely around in a full circle. Pause. Then turn a full circle in the other direction.
0. Able to turn 360° safely in 4 s or less
1. Able to turn 360° safely one side only in 4 s or less
2. Able to turn 360° safely but slowly
3. Needs close supervision or verbal cueing
4. Needs assistance while turning
8. Body sway (from UMSARS Part II–13)
0. Normal.
1. Slight body sway and/or retropulsion with unaided recovery.
2. Moderate body sway and/or deficient postural response; might fall if not caught by examiner.
3. Severe body sway. Very unstable. Tends to lose balance spontaneously.
4. Unable to stand without assistance.

*UMSARS* Unified Multiple System Atrophy Rating Scale, *SARA* Scale for the Assessment and Rating of Ataxia, *BBS* Berg Balance Scale

## Methods

The present prospective observational study included hospitalized patients and outpatients receiving treatment in the Departments of Neurology at Hokkaido University Hospital and Obihiro Kosei Hospital between January 1, 2014 and February 28, 2015. Included patients had been diagnosed with probable or possible MSA per criteria defined in the 2008 consensus statement [10]. The present study was approved by the institutional review board of Hokkaido University Hospital. Written informed consent was obtained from all patients prior to their participation in the study. Those who declined to participate as well as those with severe cognitive impairments such as inability to understand explanations or to follow instructions in examination were excluded.

Previous reports utilizing both SARA and BBS were consulted in the design of the present study [11, 12]. Patients were separately evaluated by two independent neurologists. Patients first underwent evaluation by Rater 1 using the UMSARS, SARA, and pilot scale. Rater 2 evaluated patients using the pilot scale alone on the same day. Within one month, patients underwent re-evaluation by Rater 1 using the pilot scale. Each trial was performed blindly, under the same conditions, and in avoidance of acute phases in order to eliminate the influence of sudden changes in symptoms. No interventions were utilized in the present study, and patients were allowed to continue treatments (mainly drug and rehabilitation) already in progress. Amassed data were subjected to linkable anonymizing, following which statistical analyses were performed.

### Statistical analysis

JMP® Pro Version 12.0.1 (SAS Institute Inc., Cary, NC, USA) was used for statistical analysis. Correlations between scores on the UMSARS, SARA, and the pilot scale were evaluated by Spearman’s rank coefficients. Inter-rater and intra-rater reliability for the pilot scale was assessed between Rater 1 and Rater 2. The total score as well as individual item scores for the pilot scale were analyzed based on Cronbach’s α coefficients and intraclass correlation coefficients (ICCs). Items with Cronbach’s α coefficients of more than 0.8 were considered to exhibit high internal consistency. ICCs were interpreted in conformity to the reference as slight (0.000 to 0.200), fair (0.201 to 0.400), moderate (0.401 to 0.600), substantial (0.601 to 0.800), or almost perfect (0.801 to 1.000) [13]. Mean values were presented along with standard deviations (SD).

## Results

**Fig. 1:**
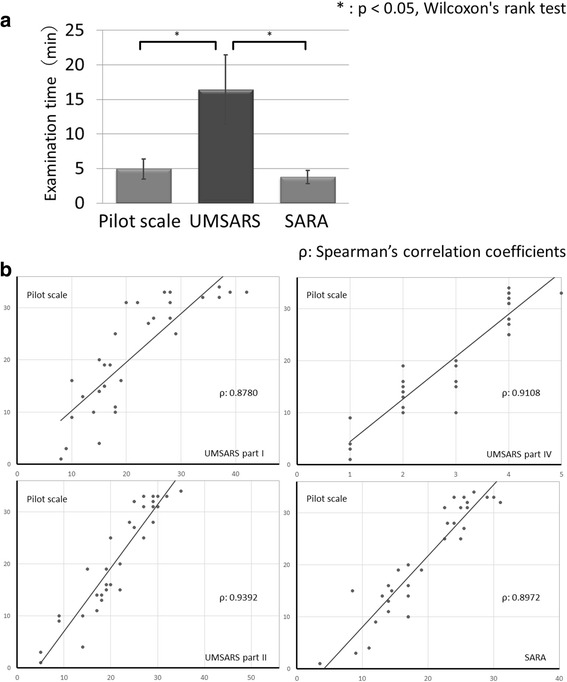
Comparison of scales. **a**The average time required for examination of UMSARS, SARA and the pilot scale. SARA and the pilot scale needed shorter time for examination than UMSARS. UMSARS, Unified Multiple System Atrophy Rating Scale; SARA, Scale for the Assessment and Rating of Ataxia. *: *p* < 0.05, Wilcoxon’s rank test. **b**. Correlation between scale scores. The scores on the pilot scale significantly correlated with those on UMSARS Parts I, II, IV and SARA. UMSARS, Unified Multiple System Atrophy Rating Scale; SARA, Scale for the Assessment and Rating of Ataxia. ρ: Spearman’s correlation coefficients

A total of 32 patients (15 male, 17 females; mean age: 63.4 ± 9.7 years old; range: 41 to 80 years old) were enrolled in the present study. Demographic information for included patients is presented in Table 2. Twenty patients had been diagnosed with MSA of the cerebellar subtype [MSA-C], while 12 patients had been diagnosed with MSA of the parkinsonian subtype [MSA-P]. The average time required for assessment was 16.4 ± 5.2 (range: 11–25) minutes for the UMSARS, 3.8 ± 1.0 (2–6) minutes for the SARA, and 5.0 ± 1.5 (2–7) minutes for the pilot scale (Fig. 1a).

**Table 2:** The characteristics of the study patients

	Total	MSA-P	MSA-C	p
Patients, n	32	12	20	
Gender, female (%)	17 (53)	6 (50)	11 (55)	
Onset age, years, mean ± SD	59.9 ± 9.4	64.3 ± 7.3	57.2 ± 9.7	*
Disease duration, years, mean ± SD	3.6 ± 2.0	2.9 ± 2.0	4.0 ± 1.9	
Probable MSA, n	27	9	18	
Scale scores (range), mean ± SD				
UMSARS part I (0–48)	21.3 ± 9.3	21.0 ± 7.7	21.5 ± 10.3	
UMSARS part II (0–56)	21.3 ± 7.8	22.9 ± 6.5	20.3 ± 8.5	
UMSARS part III (systolic decrease)	25.8 ± 17.0	26.1 ± 15.0	25.6 ± 18.5	
UMSARS part III (diastolic decrease)	13.3 ± 13.2	12.5 ± 14.0	13.7 ± 13.1	
UMSARS part IV (1–5)	3.0 ± 1.2	2.9 ± 1.1	3.1 ± 1.2	
SARA (0–40)	19.3 ± 6.9	17.5 ± 6.1	20.4 ± 7.4	
Pilot scale (0–36)	20.8 ± 10.2	20.2 ± 9.8	21.2 ± 10.7	

*UMSARS* Unified Multiple System Atrophy Rating Scale, *SARA* Scale for the Assessment and Rating of Ataxia

*: *p* < 0.05, Wilcoxon’s rank test

Total scores on each scale are presented in Table 2. Average scores for the first assessment were as follows: UMSARS Part I: 21.3/48, UMSARS Part II: 21.3/56, UMSARS Part IV: 3.0/5, SARA: 19.3/40, pilot scale: 20.8/36. There was no significant difference in the total score of the pilot scale between MSA-C and MSA-P (average total score of MSA-C: 21.2, MSA-P: 20.2). The same thing was also confirmed for each item’s score. Both total and individual item scores on the pilot scale significantly correlated with scores on UMSARS Parts I, II, and IV as well as SARA scores (Fig. 1b). Spearman’s correlation coefficients ρ were 0.8780–0.9392. No significant differences were observed between each assessment of the pilot scale (Wilcoxon’s rank test: *p* = 0.898 to 0.973). Table 3 depicts the distribution of scores assigned by Rater 1 during the first assessment. Scores for the second and third assessments showed similar tendencies. Many items had high item-total correlation coefficients (Spearman’s correlation coefficients: 0.525 to 0.937). ICCs and Cronbach’s α coefficients are presented in Table 4. Inter-rater and intra-rater ICCs and Cronbach’s α coefficients for total pilot scores were both greater than 0.9. Further, inter-rater and intra-rater ICC values over 0.6 (substantial) were obtained for almost all items on the pilot scale: Only item 2 exhibited a moderate inter-rater ICC. Cronbach’s α coefficients were greater than 0.9 for all items.

**Table 3:** Distribution of scores for rater 1 (first assessment) for the pilot scale (*n* = 32)

score	0	1	2	3	4	5	6	7	8
Items									
1. Handwriting	1	13	6	6	6	-	-	-	-
2. Finger taps	6	13	9	4	0	-	-	-	-
3. Transfers	6	12	4	6	4	-	-	-	-
4. Standing with feet together	2	6	3	6	15	-	-	-	-
5. Turning trunk	9	4	4	3	12	-	-	-	-
6. Turning 360 degree	3	0	8	4	17	-	-	-	-
7.Gait	1	0	2	8	4	1	2	8	6
8. Body sway	3	4	9	5	11	-	-	-	-

**Table 4:** Reliability of the pilot scale

Items	Intra-rater		Inter-rater	
	ICC	Cronbach α	ICC	Cronbach α
1. Handwriting	0.774	0.941	0.772	0.945
2. Finger taps	0.771	0.954	0.457	0.961
3. Transfers	0.891	0.930	0.881	0.938
4. Standing with feet together	0.876	0.930	0.827	0.935
5. Turning trunk	0.836	0.927	0.732	0.937
6. Turning 360 degree	0.917	0.931	0.861	0.938
7.Gait	0.954	0.933	0.916	0.940
8. Body sway	0.909	0.977	0.920	0.935
8 items total	0.970	0.942	0.959	0.949
7 items (excluded item 1) total	0.972	0.941	0.952	0.945
7 items (excluded item 2) total	0.966	0.954	0.958	0.961
7 items (excluded item 5) total	0.973	0.927	0.966	0.937
6 items (excluded item 1 & 2) total	0.967	0.958	0.949	0.962
6 items (excluded item 1 & 5) total	0.973	0.923	0.961	0.929
6 items (excluded item 2 & 5) total	0.971	0.942	0.968	0.952
5 items (excluded item 1, 2 & 5) total	0.969	0.947	0.962	0.953

*ICC* intraclass correlation coefficient

Additionally, we considered prototype pilot scales consisting of five to seven items by excluding either a single item or a combination of three items (item 1: hand writing, item 2: finger taps, item 5: turning trunk) with relatively low inter-rater ICCs from the original pilot scale. Exclusion of such items maintained high total scores, intra-rater and inter-rater ICCs, and Cronbach’s α coefficients.

## Discussion

Patients in the present study exhibited characteristics similar to those reported in previous studies of Asian/Japanese populations (Table 2) [14–16]. The distribution of UMSARS Part IV scores indicated that this study included relatively unbiased patients with mild to severe symptoms.

Scores on the pilot scale significantly correlated to scores obtained on the UMSARS and SARA (Fig. 1b), indicating the criterion-related validity of the pilot scale. The ability to administer this pilot scale in a short period of time further suggests its usefulness in the evaluation of MSA symptoms (Fig. 1a). In addition, ICC and Cronbach α coefficients remained high (Table 4), indicating high intra- and inter-rater reliability. Test-retest reliability and internal consistency were also high. When either one or three low inter-rater ICC items were excluded from pilot scale (Table 4), ICCs and Cronbach’s α coefficients remained relatively unchanged, indicating that a scale consisting only of items related to gait/standing is equally useful in assessing symptoms of MSA.

The present study possesses some limitation. Pilot scale items with low inter-rater ICC (handwriting, finger taps, turning trunk) exhibited ambiguity with respect to differentiating between scores. Further improvement in these areas of evaluation is required in order to more accurately assess changes in MSA symptoms. One such possibility involves combined assessment utilizing both the pilot scale and a gait accelerometer to record quantitative data. In addition, semi-quantitative scales such as that utilized in the present study often exhibit a ceiling effect [17]. This pilot scale also might show a ceiling effect among patients of advanced stage. Then, this pilot scale was not suitable for advanced MSA patients. On the other hand, in clinical trials, many participants would be early cases with mild to moderate symptoms, so influences of a ceiling effect were thought to be less likely. Further investigation regarding this point is required to more fully examine the effect of time course on the utility of the pilot scale. Additionally, this study included patients with mild to severe symptoms of MSA. And MSA-P patients were indeed relatively few. It is desirable that the reliability of this pilot scale would be presented in a larger cohort.

The SARA score of MSA-C group was similar to that of MSA-P group in this study. It should be noted that the score of SARA may be influenced by other symptoms such as Parkinsonism. The pilot scale of this study reflected symptoms of Parkinsonism and ataxia. It can be applied equally in both group without any modification. And the pilot scale showed larger standardized response mean than SARA and UMSARS [4]. It meant the pilot scale could sensitively capture symptom changes among MSA patients. The pilot scale was superior to SARA in terms of sensitivity even if it took some more time (5.0 ± 1.5 min) than SARA (3.8 ± 1.0 min). It is useful if it can suppress the deterioration of items with rapid symptomatic change (= items of this pilot scale) in clinical trials.

## Conclusions

The results of the present study indicate that the eight-item pilot scale for the assessment of MSA symptoms is both valid and reliable and may be useful for evaluation of patients in the early stages of MSA. However, due to the limitations of the present study and small sample size, further research involving improved scales as well as larger patient populations is required.

## Abbreviations

BBS: Berg Balance Scale
ICC: Intraclass correlation coefficient
MSA: Multiple system atrophy
MSA-QoL: MSA Health-Related Quality of Life scale
SARA: Scale for the Assessment and Rating of Ataxia
SCOPA-AUT: Scales for Outcomes in Parkinson’s Disease–Autonomic Questionnaire
UMSARS: Unified Multiple System Atrophy Rating Scale
